# Detection of Protein Markers From Blood Samples of Cervical Cancer Patients

**DOI:** 10.7759/cureus.72365

**Published:** 2024-10-25

**Authors:** Shahana Sharmin, Maha Jamiruddin, Mohd. Raeed Jamiruddin, Abul Bashar Mir Md K Islam, Chowdhury R Ahsan, Mahmuda Yasmin

**Affiliations:** 1 Microbiology, University of Dhaka, Dhaka, BGD; 2 Pharmacy, Bangladesh Rural Advancement Committee (BRAC) University, Dhaka, BGD; 3 Genetics, University of Dhaka, Dhaka, BGD

**Keywords:** carcinoembryonic antigen (cea), cervical cancer, cytokeratin fragment (cyfra) 21-1, high mobility group box-1 (hmgb1), human papillomavirus (hpv), serum biomarker, sodium dodecyl sulfate-polyacrylamide gel electrophoresis (sds-page), squamous cell carcinoma antigen (scca)/ serpinb3, western blot

## Abstract

Objective

Human papillomavirus (HPV) is the prevalent cause of cervical cancer in females worldwide, necessitating the development of fast and reliable diagnostic methods for early detection of HPV. The study aims to detect serum proteins like squamous cell carcinoma antigen (SCCA/SerpinB3), cytokeratin fragment antigen 21-1 (CYFRA 21-1), carcinoembryonic antigen (CEA), and high mobility group box chromosomal protein 1 (HMGB1) as biomarkers and their combination concerning the type of HPV.

Methods

Serum samples from a total of 36 cervical cancer patients were initially subjected to sodium dodecyl sulfate-polyacrylamide gel electrophoresis (SDS-PAGE), followed by western blotting with anti-Serpin B3/SCCA, anti-CEA, anti-HMGB, and anti-CYFRA 21-1. The frequency of samples for each protein was obtained. In the Chi-square test, correlations with p-values less than 0.05 were deemed statistically significant.

Results

Irrespective of HPV type, the CEA had the highest percentage of definite responses (58.33%). Subsequently, SCCA, HMGB1, and Cytokeratin-19 protein presented 47.22%, 27.78%, and 25% responses, respectively. HPV types influenced the difference in protein combinations, with HPV-16 presenting the most positive responses followed by HPV-(16+18). HPV-18 presented the least number of affirmative responses.

Conclusion

Our study presents the CEA protein as a possible biomarker and HPV-16 as the most prominent HPV type to different parallel combinations of serum proteins. The protein combinations can be applied to future cancer detection and therapy.

## Introduction

The most common oncogenic tumor of the female reproductive system, known as cervical carcinoma, continues to be the principal reason for cancer-related mortality globally in women. The age-standardized incidence rate of 10.6 and the death rate of 6.67 per million individuals were found to be associated with about 8,268 new occurrences of cervical cancer in 2020 [1]. In Bangladesh, cervical cancer is the second most prevalent form of cancer among females aged 15 to 44 in particular [2].

Chronic sexually transmitted infections by a class of double-stranded DNA viruses known as human papillomaviruses (HPVs) are the primary cause of nearly all cervical cancer cases [3]. Numerous high and low-risk genotypes of HPV, including 16, 18, 31, 33, 35, 39, 45, 51, 52, 56, 58, 59, and 68, have been classified as causing cervical carcinogenesis [4]. The WHO Summary Report 2023 estimated that the prevalence of cervical HPV-16/18 infection among females in the Southern Asia general population at any given moment is about 4.4%, and HPVs 16/18 contribute to 80.3% of invasive cervical cancers [2].

Regardless of current multimodality management, nearly 30% of the International Federation of Gynecology and Obstetrics (FIGO) stage IB2 to stage IV patients will eventually have recurrence and metastasis in the local cervix, retroperitoneal lymph node, and pelvic wall [5,6]. Other than local relapse from an organized lymphocyst or radiation reaction, it is challenging to differentiate between those conditions. Early screening of tumor markers in asymptomatic patients with recurrence can improve the prognostic prediction and diagnostic specificity of recurrent cervical cancer, hence prolonging the overall survival [7,8].

The lack of evidence substantiating the significant correlation between biomarkers and recurrence instances has prompted the need to identify novel serum tumor markers for recurrent cervical squamous cell carcinomas (CSCC). Recent research has identified multiple nuclear proteins such as squamous cell carcinoma antigen (SCCA/SerpinB3), high mobility group box chromosomal protein 1 (HMGB1), carcinoembryonic antigen (CEA), and cytokeratin fragment antigen 21-1 (CYFRA 21-1), control the different signal transduction pathways entailed directly or indirectly in growth regulation mechanisms of disease tissues, tumor development, and cervical carcinogenesis [9-12].

Copious studies on the "classical" biomarker, namely, CEA, showed involvement in the immune response, cellular contact, cell adhesion, anoikis resistance, and liver metastasis promotion [10]. Determination of serum levels of circulating CEA by enzyme-linked immunosorbent assay (ELISA) tests has assisted in assessing colorectal, lung, gastric, pancreatic, and breast cancer progression. The increased expression after surgical resection is commonly predictive of tumor recurrence for colorectal cancer or metastasis to the lung or the liver [13]. Additionally, a biomarker belonging to the endogenous protease inhibitors family known as Serpin has been shown to function as molecular chaperones, hormonal transporters, and inhibitors of serine proteases and caspases or papain-like cysteine proteases [11]. The elevated expression of SCCA observed in chronic inflammatory diseases, squamous cell carcinomas, and adenocarcinomas of various organs is usually detected using enzyme-linked immunosorbent assay (ELISA), radioimmunoassay, western blot (WB), immunoluminometric assay, and immunohistochemistry (IHC) [11,14]. Several researchers have found that numerous factors, such as volume and size of a tumor, primary tumor invasiveness or recurrence, distant and lymph node metastasis, and immunosurveillance impairment, influence the detection of SCCA levels in serum [14,15].

CYFRA 21‑1, a circulating cytokeratin 19 soluble fragment owing to necrosis of epithelial tumor cells, has been demonstrated to be one of the promising biomarkers for the prognosis of breast, non‑small‑cell lung, esophageal, gastric, and pancreatic carcinomas [16]. Numerous tumor types, including hepatocellular carcinoma, breast carcinoma, prostate cancer, colorectal cancer, and pancreatic cancer, have also been shown to overexpress HMGB1 [17]. In addition to its extracellular role as an inflammatory cytokine, HMGB1 is implicated intracellularly in nuclear and cytoplasmic functions such as telomere maintenance, DNA replication and repair, transcriptional regulation, nucleosome assembly, and autophagy [12,18]. In several cancer types, HMGB1 is associated with a poor prognosis and participates in the regulation of the development of metastases. Combined SerpinB3, HMGB1, and CYFRA 21-1 measurements improved the diagnostic sensitivity [7].

Our study identifies the significance of CEA, HMGB1, SCCA, and CYFRA 21-1 proteins individually and combined as biomarkers for early cervical cancer diagnosis to improve the patient's survival rate. We have also focused on detecting the expression levels of each serum nuclear protein in combination with different HPV genotypes using SDS-PAGE followed by western blotting techniques.

## Materials and methods

Serum samples were obtained between January 2018 and November 2019 from a total of 36 patients with HPV-positive cervical carcinoma staged clinically using the FIGO staging criteria [5,6] to examine the expression of CEA, HMGB1, CYFRA 21-1, and SCCA proteins. The serum extracted by centrifugation at 1500g for 10 minutes at 4°C was aliquoted and kept at -80°C for further investigations. The protocols were approved by the institutional review boards of Bangabandhu Sheikh Mujib Medical University, Shahbag, Dhaka (No. BSMMU/2017/151) and the Ethical Clearance Committee of the University of Dhaka (Ethical Clearance No. 30/Bio.Fac./2016-2017). Written informed consents were obtained from the patients.

Protein separation of HMGB1, SCCA, CYFRA, and CEA proteins by SDS-PAGE

Proteins were initially separated from the serum of cervical cancer patients using SDS-PAGE (sodium dodecyl sulfate-polyacrylamide gel electrophoresis), in which SDS lyses cells and denatures proteins before applying a negative charge. The aliquoted serum samples (20µg of purified protein) were added to 6x Laemmli SDS sample buffer (Sigma-Aldrich, USA), containing 20% glycerol, 50mM Tris (pH 6.8), 5% beta-mercaptoethanol, 10% w/v sodium dodecyl sulfate (SDS) (Sigma-Aldrich, USA), and 0.1% bromophenol blue, and heated at 100℃ for 5 minutes. Then, the protein lysates were applied to a 12% SDS-polyacrylamide gel and run for 1-2 hours at 100V. Later, per the manufacturer's directions, the proteins were moved from the gel onto the nitrocellulose membrane. The membrane was then stained to check whether any protein had been transferred.

Western blotting of HMGB1, SCCA, CYFRA, and CEA proteins

The nitrocellulose membrane was then blocked by incubating it with 5% skimmed milk and TBS buffer (Sigma-Aldrich, USA) at room temperature for 1 hour. Subsequently, the membrane was incubated for 1 hour with the following antibodies: anti-Transferrin antibody (1:1000; Abcam, ab82411, USA) as an internal control; anti-HMGB1 antibody (1:1000; Abcam, ab18256, USA); anti-CEA antibody (1:500; Abcam, ab33562, USA); anti-Cytokeratin 19 antibody (1:300; Abcam, ab53119, USA); and anti-SerpinB3/SCCA antibody (1:1000; Abcam, ab154971, USA). Following the incubation, the membrane was washed threefold using tris-buffered saline with tween 20 (TBST) for 5 minutes each. Then, the membrane was incubated with HRP-conjugated goat anti-rabbit IgG antibody (1:2000; Abcam, ab205718, USA) at room temperature for 1 hour, followed by three 5-minute TBST washes. Finally, the antigen-antibody complex was visualized by staining the membrane with diaminobenzidine (DAB) (Abcam, USA). The molecular weight of the following proteins is: transferrin is 77 kDa, SCCA/SerpinB3 protein is 45 kDa, CYFRA 21-1/cytokeratin 19 fragment is 44 kDa, CEA is 77 kDa, and HMGB1 antigen is 29 kDa.

Statistical analysis

All the experimental data were analyzed using the Chi-square test, and the correlations with p-values <0.05 were regarded as statistically noteworthy.

## Results

Patient classification based on cancer biomarker

In this study, four distinct kinds of multifunctional, redox-sensitive proteins were employed as antibodies against the four different protein types that were considered antigens. Table 1 below shows the age distribution of Figo stages with HPV genotypes of the 36 patients included in this study.

**Table 1:** The age distribution of FIGO stages with HPV genotypes of the 36 cervical cancer patients FIGO: International Federation of Gynecology and Obstetrics; HPV: Human papillomavirus

Patient's age range (in years)	FIGO Stages	HPV Types			
		Type-16	Type-18	Type-(16+18)	Others
31-40	I	0	0	0	0
	IA	1	0	0	0
	IIA	1	0	0	0
	IB	2	1	1	1
	IIB	0	1	0	0
41-50	I	0	0	1	0
	IA	3	0	6	0
	IIA	1	0	0	0
	IB	3	1	1	0
	IIB	0	0	1	0
51-60	I	0	0	1	0
	IA	2	0	0	0
	IIA	1	0	1	0
	IB	0	1	1	0
	IIB	4	0	0	0

**Figure 1:**
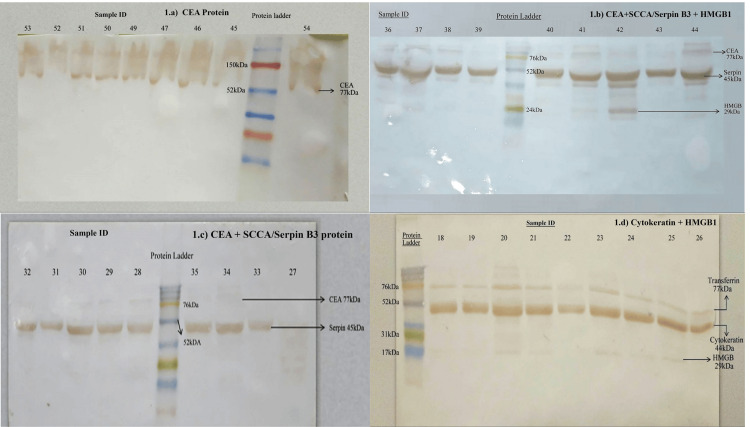
Expression of cancer biomarker a) Western blotting of individual's serum (45 - 54) using Serpin B3, HMGB1, and CEA antibody where samples were found to be positive for CEA protein only; b) Western blotting of individual's serum (36 - 44) using Serpin B3, HMGB1, and CEA antibody where samples were found to be positive for CEA, Serpin B3, and HMGB1 protein; c) Western blotting of individual's serum (27 - 35) using Serpin B3, HMGB1 and CEA antibody where samples were found to be positive for CEA and Serpin B3 protein; and d) Western blotting of individual's serum (18 - 26) using Cytokeratin and HMGB1 antibodies, and Transferrin antibody as an internal control where the samples were found to be positive for both Cytokeratin and HMGB1 protein. SCCA/SerpinB3: Squamous cell carcinoma antigen; CYFRA 21-1: Cytokeratin fragment antigen 21-1; CEA: Carcinoembryonic antigen; HMGB1: High mobility group box chromosomal protein 1; HPV: Human papillomavirus

The expression of four different biomarkers, namely HMGB1, SCCA/SerpinB3, CEA, and CYFRA 21-1/Cytokeratin 19, in the serum of 36 cervical cancer patients was interpreted upon SDS PAGE and western blotting (Figure 1). In Figure 1a, the individual's serum blotted with CEA, HMGB1, and SerpinB3 antibodies showed that eight samples, except a single patient, tested positive for CEA protein only. Whereas from Figure 1b, where the serum of patients was blotted with CEA, HMGB1, and SerpinB3 antibodies, we observed that all nine samples showed bands for SerpinB3 protein, eight samples for CEA protein, and five samples for HMGB1 protein. In Figure 1c, the serum of patients was also blotted with HMGB1, CEA, and Serpin B3 antibodies. Five samples were positive for CEA protein, and eight tested positive for SerpinB3 protein. However, in Figure 1d, all nine serum samples blotted using cytokeratin and HMGB1 antibodies, with a transferrin antibody as an internal control showed the presence of cytokeratin protein, and five samples showed bands for HMGB1 protein.

Western blotting results of the cancer biomarkers

The various parallel protein combinations would aid in cancer detection. It was found that the positive responses of numerous types of proteins against various HPV types were statistically significant (p-value <0.0001). Of all the combinations of proteins, most affirmative responses were against HPV type 16 only, followed by HPV type 16+18. Then, a small number of responses against HPV type 18 were observed. On the other hand, it was also noticed that among all four proteins in the individual's serum, CEA was the notable protein that exhibited the most favorable response (58.33%; n=21), trailed by SCCA protein (47.22%; n=17), HMGB1 protein (27.78%; n=10), and Cytokeratin 19 fragment protein (25%; n=9).

Statistical assessment of the immunoblot data

**Figure 2:**
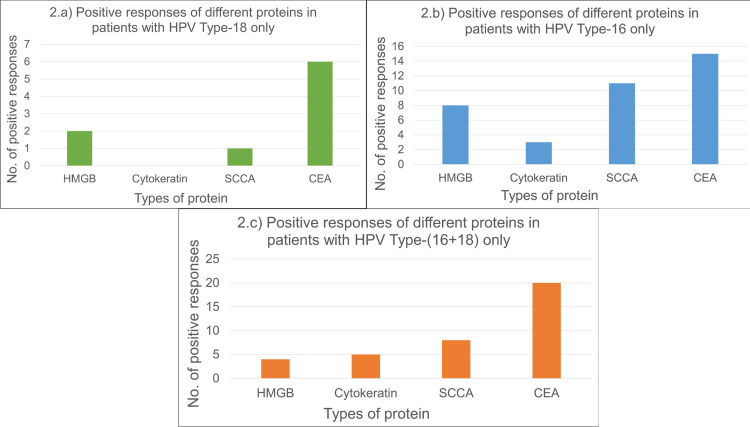
Differential expression of proteins in patients with different HPV types a) Positive responses of different proteins in patients with HPV Type-18 only; b) Positive responses of different proteins in patients with HPV Type-16 only; c) Positive responses of different proteins in patients with HPV Type-(16+18) only. SCCA/SerpinB3: Squamous cell carcinoma antigen; CYFRA 21-1: Cytokeratin fragment antigen 21-1; CEA: Carcinoembryonic antigen; HMGB1: High mobility group box chromosomal protein 1; HPV: Human papillomavirus

Figure 2, represented below, demonstrates the differential expression of the four distinct proteins (namely HMGB1, SCCA/Serpin B3, CEA, and CYFRA 21-1/Cytokeratin 19) against HPV type 16, 18, and both 16+18 in cervical cancer patients. In the chi-square test, a p-value below 0.05 was deemed statistically important to identify the quantity of each protein from the serum samples of cervical cancer patients. In HPV Type 18 only (Figure 2a), CEA protein showed six positive responses, which was the highest, then HMGB1 protein showed two positive responses, and SCCA protein showed a single positive response. Cytokeratin 19 protein did not elicit any noteworthy positive outcomes. However, in Figure 2b, the CEA protein had the highest number of positive responses of fifteen among all four proteins for HPV Type 16 only. In contrast, Cytokeratin 19 protein had the lowest number of positive results of three. Positive outcomes for HMGB1 and SCCA were eight and eleven, respectively. Furthermore, from Figure 2c, we note that individuals with HPV Type 16+18 displayed twenty confirmed responses for the CEA protein, eight for the SCCA protein, five for the Cytokeratin 19 protein, and four for the HMGB1 protein.

The assessment of the total number of positive outcomes of all four proteins from the patient's serum having HPV 16, 18, and both 16+18, CEA protein showed prominent expression in the patients, with the levels of the SCCA/Serpin B3 protein, HMGB1 protein, and Cytokeratin 19 fragment protein gradually declining.

## Discussion

In many developed countries, cytology-based screening programs that are well-organized and of high quality have significantly decreased the incidence of cervical cancer. In developing nations such as Bangladesh, the setup of cytology-based screening programs is complicated. In such cases, pap tests are required, which are costly, followed by colposcopy and biopsy as diagnostic work-up. Establishing and sustaining cytology-based programs has been challenging in low-resource countries [19]. Thus, within the last ten years, several novel biomarkers have been discovered due to advances in our knowledge of the biology of the human papillomavirus and the natural history of pre-cancerous and malignant lesions linked to the virus [7,20,21]. In the present study, the prevalence of the CEA, CYFRA 21-1 proteins, SCCA, and HMGB1 in the serum of cervical cancer patients was only regarded.

Our study shows that CEA is the most prevalent protein found in approximately 20% of all cervical cancer samples, consisting of HPV types 16, 18, and 16+18 combinations, indicating it is a potential biomarker in line with previous reports [20]. A previous CEA immunohistochemical study reported a significant positive relation between the expression of CEA and the progression stages of the SCC of the cervix [22]. Additionally, CEA was detected at varying rates of 58.1% and 16.7% in cases with and without recurrence, respectively, during pretreatment screening in individuals with cervical SCC. Therefore, CEA is a crucial tumor marker for recurrence prediction, with a median time of 13 weeks to recurrence in CEA-positive patients [23].

In patients with HPV Type-18 and HPV Type- (16+18), the SCCA/SerpinB3 protein might be a relevant biomarker. In a meta-analysis, the serum SCCA levels were found to be invariably correlated with relapse and mortality in cervical cancer individuals, assisting in the forecast of disease progression and therapeutic response [21,24]. Serum SCCA prompt response assessment is a potent predictive tool. Additionally, a prior immunohistochemical investigation that examined the expression levels of SCCA in different cervical lesion tissues revealed a progressive increase in SCCA expression levels as the degree of cervical lesion severity increased [25]. These results imply that molecular targeting of SCCA and therapy escalation should be considered in patients with elevated or persistent blood SCCA levels [26].

Though SCCA/SerpinB3 expression was absent in HPV type-16 patients, the expression of HMGB1, a nuclear DNA-binding protein, was observed in higher amounts, in addition to its overexpression in HPV type (16+18) patients. This lends credence to its significance as a potential biomarker and a differentiation factor for the kind of HPV infection in congruence with SCCA/SerpinB3. Li et al. demonstrated that the tumor size, cervical stromal invasion depth, parametrial infiltration, and FIGO stage were strongly correlated with HMGB1 upregulation, as evidenced by immunohistochemical analysis of cervical cancer tissues [27]. Hence it may be inferred that HMGB1 expression facilitates metastasis and proliferation of cervical cancer cells, thereby serving as a valuable prognostic factor and possible biomarker for cervical cancer. In a univariate analysis, a significant recurrence of HPV infection is linked to elevated HMGB1 protein in cervical cancer samples [28].

A combination of protein expressions could be presented as a diagnostic tool for the early detection of cervical cancer and patient prognosis assessment. SCCA typically serves as a cervical cancer prognosticator and predictor. Previous studies examined the relationship between serum SCCA and HMGB1 expression [7]. SCCA and HMGB1 expression were positively correlated, suggesting that HMGB1 may serve as a biomarker for assessing the biological actions and prognosis of cervical cancer [29].

Assessment of proteins, enzymes, and metabolites can help create more effective biomarkers for the timely diagnosis and treatment of gynecological cancers, particularly cervical cancer [30]. Thus far, it has been observed that combinations of various protein analyses may be able to validate cervical cancer. For monitoring disease recurrence and predicting prognosis in cervical squamous cell carcinoma patients, serum HMGB1 levels may be a helpful and precise diagnostic. The diagnostic specificity and sensitivity were improved by combined assessments of SCCA, HMGB1, and CYFRA 21-1 serum levels [7].

Our study does present some limitations, such as the lack of protein quantification at various levels and the correlation of protein expression with the prognosis of the disease. A tumor marker is deemed optimal when it offers information on tumor burden and activity, as well as enough adequate demarcation with high specificity and sensitivity to distinguish between individuals with cancer and healthy controls or those with benign diseases.

## Conclusions

Our study presented the CEA protein as a potential biomarker for cervical cancer. Moreover, SCCA, HMGB1, and CYFRA 21-1 may constitute a pivotal point of cervical oncogenesis, presenting them as prospective targets for cervical cancer prognosis. Additionally, HPV-16 was the most prominent HPV type in different parallel combinations of serum proteins. Nevertheless, before a definitive conclusion is made, additional research using a large prospective trial or a meta-analysis of small-scale retrospective or prospective studies can validate the therapeutic relevance of the blood tumor markers. Currently, there are few approved tumor markers for use in cervical cancer patient follow-up, prognostic assessment, treatment monitoring, or diagnosis. In the coming years, it may be anticipated that primarily HPV screening programs and secondarily novel biomarkers testing shall be put into action for the early detection of women's risk of cervical cancer rather than tending to any high-grade lesion. The biomarkers described in this study could be crucial in guiding future therapeutic choices.
